# Geospatial variation and machine learning approaches to predict open defecation practice in Zambia

**DOI:** 10.1371/journal.pone.0350923

**Published:** 2026-06-10

**Authors:** Andualem Addisu Birlie, Nega Abebe Meshesha, Sefefe Birhanu Tizie, Smegnew Gichew Wondie, Selamawit Gashaw Teshome, Tesfaye Deribe Bedada, Ayana Alebachew Muluneh, Biruktawit Lelisa Eticha, Geleta Nenko Dube, Gelgelo Wodessa, Muluken Belachew Mengistie

**Affiliations:** 1 Department of Health Informatics, College of Medicine and Health Science, Samara University, Samara, Ethiopia; 2 Department of Public Health, Institute of Health, Dambi Dollo University, Dambi Dollo, Ethiopia; 3 Department of Health Informatics, College of Medicine and Health Science, Debre Markos University, Debre Markos, Ethiopia; 4 Department of Public Health, College of Medicine and Health Science, Mizan Tepi University, Mizan Aman, Ethiopia; 5 School of Public Health, Health Science Campus, Debre Berhan University, Debre Berhan, Ethiopia; 6 Department of Health Informatics, School of Public Health, Institute of Health, Bule Hora University, Bule Hora, Ethiopia; 7 Department of Health Informatics, College of Medicine and Health Science, Wollo University, Dessie, Ethiopia; 8 Department of Health Informatics, School of Public Health, College of Medicine and Health Science, Wachemo University, Hossana, Ethiopia; 9 School of Public Health, Institute of Health, Bule Hora University, Bule Hora, Ethiopia; National Research and Innovation Agency, INDONESIA

## Abstract

**Background:**

Open defecation is the disposal of human feces in fields, bushes, forests, open waterways, beaches, and other open areas. It worsens the environment, contaminates drinking water sources, causes malnutrition and low school attendance in children, and aids in the spread of diseases like cholera, diarrhea, dysentery, typhoid, polio, and hepatitis A. The purpose of this study was geospatial variation and machine learning approaches to predict open defecation in Zambia.

**Methods:**

This study used secondary data analysis from the cross-sectional Zambia Demographic and Health Survey (ZMDHS) 2024. Spatial distribution, spatial autocorrelation, incremental autocorrelation, spatial interpolation, and hot spot area detection were all examined using ArcGIS 10.7. Python was used to identify the features of open defecation practice using machine-learning algorithms. We carried out an 80/20% data split, one-hot data encoding, data transformation and integration, data cleaning, and 10-fold stratified cross-validation. This study employed seven machine-learning algorithms, including adaptive boosting, cat boosting, random forest, light boosting, extreme gradient boosting, decision tree and logistic regression.

**Results:**

Among 12,808 households in Zambia, 12.1% were practiced open defecation. Spatial analysis revealed significant clustering, with hot spots concentrated in Southern, Western and Eastern regions, highlighting areas in urgent need of intervention. Machine learning models were applied to predict open defecation practices, with light gradient boost performing the best model with AUC of 83.83%. From this study, 198 true positives were generated by the model for the classification of open defecation practice, accurately identifying those who reported engaging in this behavior.

**Conclusion:**

Wealth index, access to treated water, access to electricity, educational level, and age of household head, access to media, and region were the most significant features of open defecation practice. Governments, NGOs, policy makers, and researchers can use these data to create targeted interventions for improving health and environmental sanitation based on the gaps and disparities discovered.

## Background

Open defecation is the term used to describe the disposal of human feces in fields, bushes, forests, open waterways, beaches, and other open areas [1]. Developing countries, particularly those in Asia and Sub-Saharan Africa, are disproportionately affected by the dangerous and damaging practice of open defecation, which also contributes to pollution [2]. It poses environmental and social problems and aids in the spread of diseases, making it a serious public health concern [3]. Poor sanitation causes the deaths of 775,000 people worldwide each year [4]. 2.4 billion people worldwide, the majority of whom reside in Sub-Saharan Africa (SSA), lacked access to improved sanitation in 2015 [5]. In 2020, World Health Organization (WHO) and United Nations Children’s Fund (UNICEF) reported that over 673 million people worldwide engage in open defecation, and approximately 3.6 billion people lack access to safely managed sanitation services [4]. 494 million people engage in open defecation, according to WHO and UNICEF Joint Monitoring Program (JMP) 2021 reports [6]. Nearly half of them were from Sub-Saharan Africa (SSA), and the majority (92%) of them lived in rural areas. The environment, the economy, and societal health are all negatively impacted by inadequate sanitation facilities and poor sanitation [4,7]. It worsens the environment, contaminates drinking water sources, causes malnutrition and low school attendance in children, and aids in the spread of diseases like cholera, diarrhea, dysentery, typhoid, polio, and hepatitis A [8,9]. Inadequate sanitation, tainted drinking water, and poor hygiene are the main causes of diarrheal illnesses worldwide, accounting for over 297,000 deaths among children under five in 2019 [6,10]. According to a study, communities that practice open defecation had a fourfold higher prevalence of diarrhea than communities that do not [11]. Additionally, there is a risk that open defecation will expose hundreds of millions of women and girls worldwide to increased sexual exploitation and a lack of privacy during their periods [12].

Research indicates that either poverty, which makes it difficult to construct latrines, or a lack of government support and enforcement of them are the main causes of open defecation. However, despite the availability of restrooms, people frequently choose to defecate outside [13]. The households’ access and utilization will be influenced by a variety of factors, including housing, environmental factors, and sociodemographic factors. The household heads’ age, sex, level of education, marital status, wealth index, number of household members, region, and number of under five children. Other potential contributing factors to the practice of open defecation included access to a water source, electricity, the internet, access to treated water, residence, and media [12–15].

Machine learning tools are known to improve the accuracy of short-term prediction and to create the best possible conditions for the advancement of sanitation management [4]. We measure and interpret the relative impact of features using SHAP analysis, which captures intricate nonlinear relationships that are frequently overlooked by traditional techniques. However, there is no evidence at the national level regarding the contributing factors to open defecation practice in Zambia using on both spatial and machine learning analysis. Understanding the variables that affect the rate of sanitation improvement and the reduction of diarrhea morbidity and mortality brought on by poor sanitation is crucial.

The purpose of this study was geospatial variation and machine learning approaches to predict open defecation in Zambia. This study uses a machine learning approach to identify important features within hot spot areas with elevated rates of open defecation. The study offers practical insights for mitigating sanitation disparities, maximizing resource allocation, bolstering climate-resilient WASH programs, and assisting policymakers in creating efficient, focused interventions by fusing data-driven prediction with geospatial analysis. Additionally, in environments with limited resources, the predictive framework provides a transferable model for environmental and health applications.

## Methods

### Study design, setting, and period

**Fig 1:**
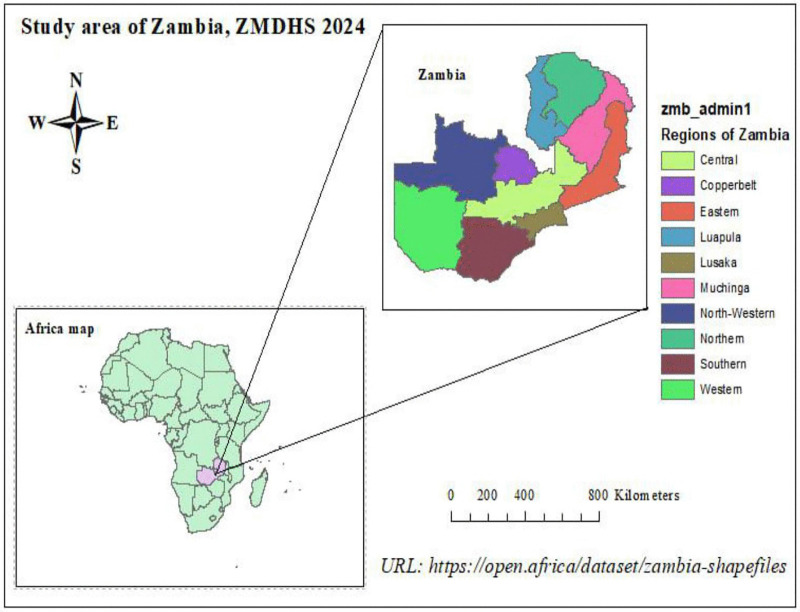
Study area of open defecation practice in Zambia‌‌, ZMDHS 2024.

This study used secondary data analysis from the cross-sectional Zambia Demographic and Health Survey (ZMDHS) 2024, which was carried out from January 8 to July 5, 2024. Zambia is found in the Eastern Africa, which has 10 regions, such as Central, Copperbelt, Eastern, Luapula, Lusaka, Muchinga, Northern, Northwestern, Southern, and Western regions as shown in Fig 1. Finally, we developed a predictive model that predicts open defecation practice in Zambia.

### Data sources, sample size and sampling technique

This study used a secondary analysis of data from the Demographic and Health Surveys (DHS). We used Household Record (HR) and other online-accessible measured DHS program data. Urban and rural enumeration districts were established for sampling purposes in each of Zambia’s ten regional states. A total sample of 12,808 households from the ten regions was included in this study using the most recent ZMDHS dataset. A two-stage stratified cluster sampling procedure was used to select study participants. In the first step, a stratified sample of enumeration areas (EAs) was selected at random. In the second stage, households within the selected EAs were selected using systematic random sampling. A tailored questionnaire was used to interview the selected household.

### Variables of the study

#### Outcome variable.

The dependent variable of this study was open defecation practice, based on WHO/UNICEF JMP [16]. The DHS data contains a variable entitled “Type of toilet facility,” so we recorded those households’ responses into two categories. Those households that used “no open defecation practice” as “0” and those households that used “open defecation practice” were coded as “1”. Open defecation practice was used to categorize households with no facility or defecating in bush or field, whereas no open defecation practice was used for those who have sanitation facilities [4,12,14,15,17].

#### Explanatory variables.

The study included different household characteristics such as the age of the household head, sex of the household head, educational level, marital status, and wealth index, number of household members, region and number of under five children. In addition, access to a water source, access to electricity, access to internet, access to treated water, place of residence, and access to media [12–15].

### Data management and statistical analysis

#### Spatial analysis.

STATA/MP version 17 and Microsoft Excel 2019 were used for data preprocessing, which included missing data handling, cleaning, recoding, and appending data. The [IW = hv005/1000000] command in STATA was used to apply sampling weights (hv005) in order to guarantee representativeness and produce accurate estimates. Spatial distribution, spatial autocorrelation, incremental autocorrelation, spatial interpolation, and hot spot area detection were all examined after the weighted percentage of open defecation data was exported to ArcGIS version 10.7 software. Kuldorff’s SaTScan version 9.6 software was used to apply spatial scan statistics.

All spatial analyses were conducted at the DHS cluster level, since Global Positioning System (GPS) coordinates are only available for clusters and are intentionally displaced to preserve confidentiality. Household records were linked to their respective cluster coordinates, and the magnitude of open defecation practice was aggregated at the cluster level prior to analysis. In total, 545 clusters from the 2024 Zambia DHS were included. For spatial autocorrelation, hot spot analysis, interpolation analysis and SaTScan procedures, we applied adaptive bandwidth spatial weights matrix to ensure optimal model performance and account for spatially variable data density.

**Incremental autocorrelation analysis:** To assess spatial autocorrelation over a range of distances, incremental autocorrelation analysis was created, displaying Z-scores and the distances that corresponded to them. Z-scores provide information about the degree of spatial clustering as well as its statistical importance. Where clustering-promoting spatial processes are most noticeable, Z-score peaks indicate the distance. These peak distances help choose the best threshold or radius by offering vital guidance for tools that incorporate distance band or distance radius parameters. Tools like hot spot analysis that depend on these parameters for efficient spatial analysis find this useful information [3].

**Spatial Autocorrelation (Global Moran’s I) analysis:** GPS coordinates and open defecation proportions were used in the spatial autocorrelation tool to determine whether the open defecation pattern under investigation was dispersed, clustered, or random using ArcGIS version 10. 7. We were able to assess the significance of that index by looking at the calculated tools’ Moran’s I index value, Z-score, and p-value. A geographical clustering of open defecation is indicated by a statistically significant positive Moran’s I value, whereas dispersion is shown by a significant negative Moran’s index, and a random distribution is shown by a value of zero [18].

**Hot spot analysis:** By calculating the Getis-ord Gi* statistics for each area, hot spot analysis was calculated to determine how spatial autocorrelation varies throughout the study location. The p-values and Z-scores were computed to see if there was significant clustering. A cold spot is denoted by low Getis-ordGi*, while a hot spot is indicated by statistical values with high Getis-ordGi* [19].

**Spatial interpolation:** It was employed to estimate the open defecation practice in unsampled areas based on data from sampled clusters [20]. This study used both deterministic and geostatistical interpolation methods and selected ordinary kriging geostatistical interpolation method as the best accurate technique due to its lowest mean predicted error (MPE) of 0.00107 and root mean square predicted error (RMSPE) of 0.4126 as shown in Table 1**.** This study applied ordinary kriging geostatistical interpolation method to predict the open defecation practice in Zambia. A spherical semivariogram model was fitted to the data, with nugget, sill, and range parameters estimated empirically. Model performance was assessed using leave-one-out cross-validation, and prediction uncertainty was quantified through kriging variance. Although household-level outcomes are binary, cluster-level proportions were treated as continuous variables, which is consistent with geostatistical interpolation assumptions.

**Table 1:** Interpolation methods comparison of open defecation practice in Zambia, ZMDHS 2024.

Interpolation methods	Parameters	
	Mean predicted error (MPE)	Root mean square predicted error (RMSPE)
Deterministic interpolation		
Inverse distance weight d	0.00231	0.4679
Geostatistical interpolation		
**Ordinary kriging**	**0.00107**	**0.4126**
Universal kriging	0.00178	0.4526
Simple kriging	0.00539	0.4948
Disjunctive kriging	0.00528	0.4939
Indicator kriging	0.00432	0.4727
Probability kriging	0.00432	0.4727

**SaTScan cluster analysis:** Using the Bernoulli model, SaTScan cluster analysis was used to find high-likelihood clusters of open defecation practices. Households that practiced open defecation were categorized as cases, and households that did not were classified as controls. Using a circular scanning window that evaluated different cluster sizes, the scan statistic found clusters with noticeably high prevalence. To assess statistical significance, Monte Carlo simulations and likelihood ratio tests were employed. High-risk areas were identified by the results, offering guidance for resource allocation and focused sanitation interventions [14].

#### Machine learning analysis.

**Fig 2:**
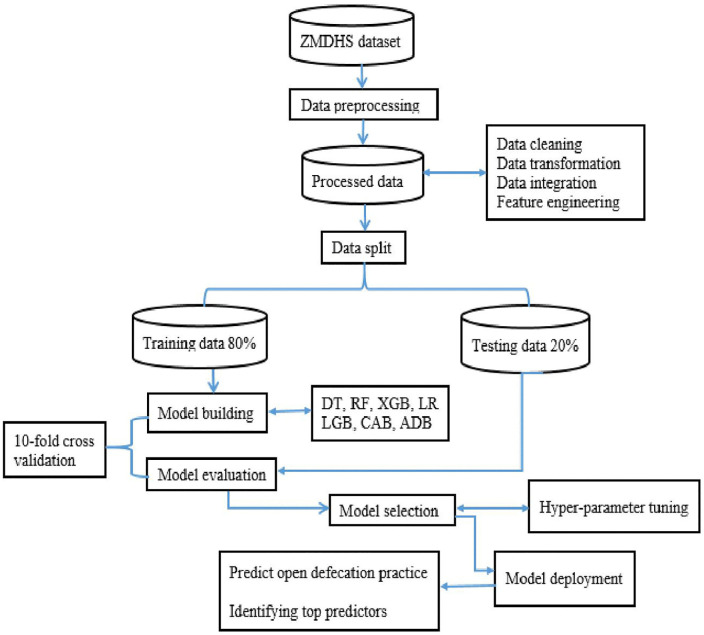
Summary of data preparation and analysis process plan for open defecation practice in Zambia, ZMDHS 2024.

Using STATA/MP version 17 and Microsoft Excel 2019, the data was extracted. It was then converted to CSV format and imported into Jupiter Notebooks’ Python (v3.12) for additional analysis. Descriptive statistics were presented as text frequencies and percentages in tables and graphs. This study uses Yufeng Guo’s 7 Steps of Machine Learning [21] as the basis for a general framework from a prior study to predict a household’s open defecation practice. Fig 2 illustrated these steps, which include data collection, preparation, model selection, training, evaluation, parameter adjustment, and prediction relevant to supervised machine learning. In this study Pandas (v2.2.2), Seaborn (v0.13.2), Scikit-Learn (v1.4.2), and SHAP (v0.50.0) were used to implement machine learning models and algorithms. Data preprocessing, transformation, integration, dimensionality reduction, splitting, discretization, and feature engineering were among the several steps in the data processing and analysis process that made sure the dataset was of high quality for machine learning predictions. The full feature set used in all models were comprised household head characteristics (age, sex, education, and marital status), number of household members and under five children, wealth index, and region, access to water, electricity, internet, treated water, media, and place of residence.

To deal with missing values, the K-nearest neighbor (KNN) imputation technique was applied. Dummy variables were made for categorical variables using one-hot encoding and the first columns from the one-hot encode variables were used for reference (comparison). Using random stratification, the entire dataset was divided into a training set (80%) and a holdout test set (20%) for data splitting and cross-validation. On the 80% training set, 10-fold cross-validation was used for all model development, hyperparameter tuning, and internal validation. Within each fold, preprocessing were applied only to the training partition to avoid data leakage, and models were evaluated on the fold’s validation partition. The Synthetic Minority Oversampling Technique (SMOTE) oversampling technique was employed to create new observations that resemble the minority class by interpolating across minority class samples in the feature space [22].

Seven supervised machine learning algorithms such as, Adaptive Boosting (AdaB), Cat Boosting (CatB), Random Forest (RF), Light Gradient Boosting (LGB), Extreme Gradient Boosting (XGB), Decision Tree (DT) and Logistic Regression (LR) were used for the model selection [23]. While ensemble approaches (RF, XGB, LGB, CatB and AdaB) were given priority due to their capacity to handle high-dimensional data, model nonlinear relationships, and prevent overfitting through bagging and boosting mechanisms [24]. Simpler models (e.g., DT and LR) were used to evaluate trade-offs in complexity [25]. The best model

The mean cross-validated performance metrics (AUC, accuracy, precision, recall, and F1 score) were used to select and interpret the performance of the model. A default probability threshold of 0.5 was used for classification of open defecation practice. For the last analysis, the model with the best predictive performance was chosen. The final model was retrained on the complete 80% training set after the best algorithm and hyperparameters were chosen, and its results were recorded on the separate 20% holdout test set. Hyperparameters were tuned using Optuna’s Bayesian optimization framework with 10-fold cross-validation. Using Shapley Additive Explanations (SHAP), which calculates the contribution of each variable to the model’s prediction, feature importance was evaluated. With this method, the relative impact of each feature on the prediction of open defecation is measured instead of depending on conventional statistical significance testing. When used in tandem, the SHAP beeswarm and bar plots provide a powerful technique for identifying relationships and patterns in the data, enhancing transparency and breaking down complex models [26].

### Ethical consideration

This study is based on secondary analysis of the publicly available Zambia Demographic and Health Survey (ZDHS) 2024 dataset. The DHS Program obtains informed consent from all participants and ensures strict confidentiality in accordance with the Declaration of Helsinki and national ethical guidelines. Since this research involved analysis of anonymized, de-identified data, no additional ethical approval was required, and Human Ethics and Consent to Participate declarations are not applicable.

## Results

### Characteristics of the respondents

**Fig 3:**
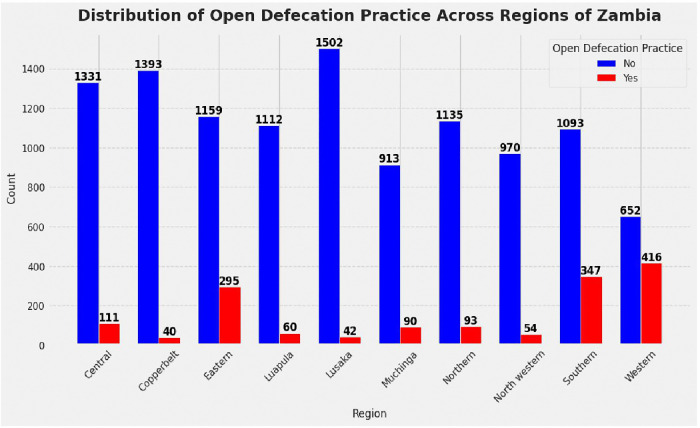
Distribution of open defecation practice in Zambia’s regions, ZMDHS 2024.

Many households among the 12,808 study participants were found in urban areas (59.11%), and most household heads were married (68.87%) and male (68.99%). Many household heads (38.27%) were between the ages of 31 and 45, and 44.84% of households were in the poor wealth category. Primary school had the highest educational attainment (42.74%). Most households had no children under five (42.83%), and most households had four to six members (44.90%). While 77.11% had access to water sources, only 34.85% used household treated water, and 70.12% lacked electricity, 72.48% lacked internet, and 46.53% had no media access. Overall, the results show that the population is mostly urban, married, and male-headed, but they continue to struggle with poverty, education, and access to basic services as shown in Table 2. Western Zambia had the highest percentage of participants who engaged in open defecation, accounting for 416 (26.9%) of the total as shown in Fig 3.

**Table 2:** Sociodemographic characteristics of the respondents for open defecation practice in Zambia ZMDHS 2024.

Variables	Categories	Frequency	Percentage
Residence	Urban	5,237	59.11
	Rural	7,571	40.89
Marital status	Never union	703	5.49
	Married	8,821	68.87
	Widowed	1,536	11.99
	Divorced	1,748	13.65
Age of household head	15-30	2,684	20.96
	31-45	4,901	38.27
	46-60	3,148	24.58
	>60	2,075	16.20
Sex of house hold head	Female	3,972	31.01
	Male	8,836	68.99
Wealth index	Poor	5,747	44.84
	Middle	2,537	19.81
	Rich	4,524	35.32
Educational level	No education	1,134	8.85
	Primary	5,474	42.74
	Secondary	4,812	37.57
	Higher	1,388	10.84
Number of household member	1-3	4,163	32.50
	4-6	5,751	44.90
	>=7	2,894	22.60
Number of under 5 children in months	0	5,486	42.83
	1	4,534	35.40
	2	2,307	18.01
	>=3	481	3.76
Access to electricity	No	8,981	70.12
	Yes	3,827	29.88
Access to internet	No	9,287	72.48
	Yes	3,525	22.89
Access to media	No	5,960	46.53
	Yes	6,848	53.47
Access to water source	No	2,932	22.89
	Yes	9,876	77.11
Access to treated water	No	8,434	65.15
	Yes	4,463	34.85

### Spatial analysis

#### Spatial incremental analysis.

**Fig 4:**
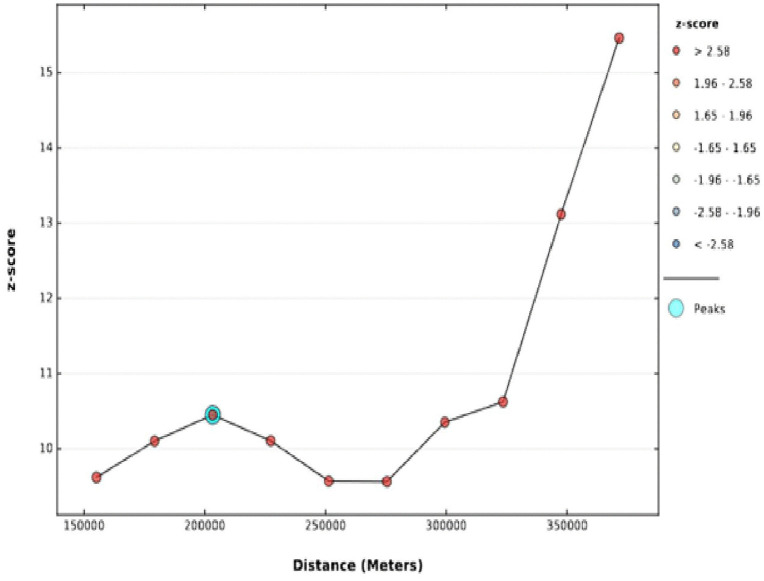
Spatial incremental analysis of open defecation in Zambia, ZMDHS 2024.

A strong influence of spatial factors that promote clustering is indicated by the presence of statistically significant Z-scores at 203,275 meters. Ten distance bands were identified by the incremental spatial autocorrelation analysis. Clustering first became noticeable at 155,193 meters and increased by 24,041 meters as shown in **Fig 4**. This implies that the frequency of open defecation is not distributed randomly but rather depends on geographic proximity.

#### Spatial autocorrelation (Global Moran’s Index) analysis.

**Fig 5:**
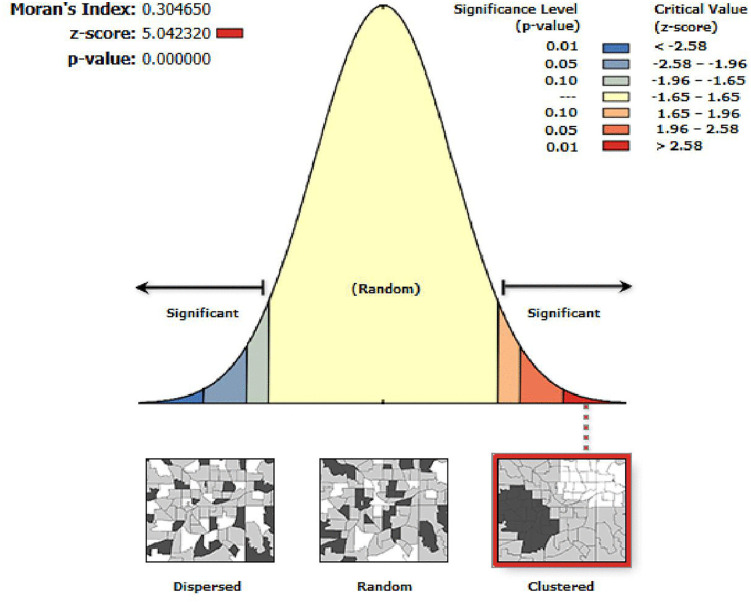
Spatial autocorrelation of open defecation in Zambia, ZMDHS 2024.

With a Z-score of 5.042 and a Moran’s Index of 0.305 (p-value less than 0.001), the spatial autocorrelation of this study showed a high and statistically significant clustering pattern of open defecation practice, suggesting that there is less than a 1% chance that this clustered pattern could be the product of random choice as illustrated in Fig 5.

#### Hot spot analysis (Getis-Ord Gi*).

**Fig 6:**
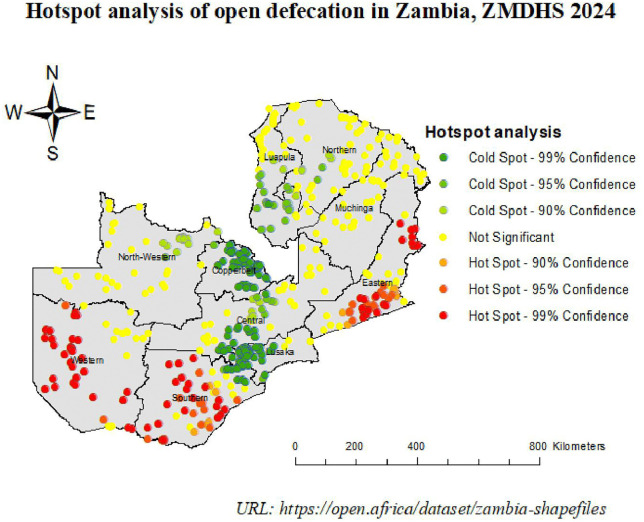
Hot spot analysis of open defecation in Zambia, ZMDHS 2024.

Hot spot analysis identified statistically significant hot spot areas of open defecation practice were observed in Southern, Western and Eastern regions of Zambia. There were statistically significant cold-spot areas of open defecation practice in the Copperbelt, Luapula, Lusaka, central and some parts of Northern and Southern of regions of Zambia presented in Fig 6.

#### Spatial interpolation analysis.

**Fig 7:**
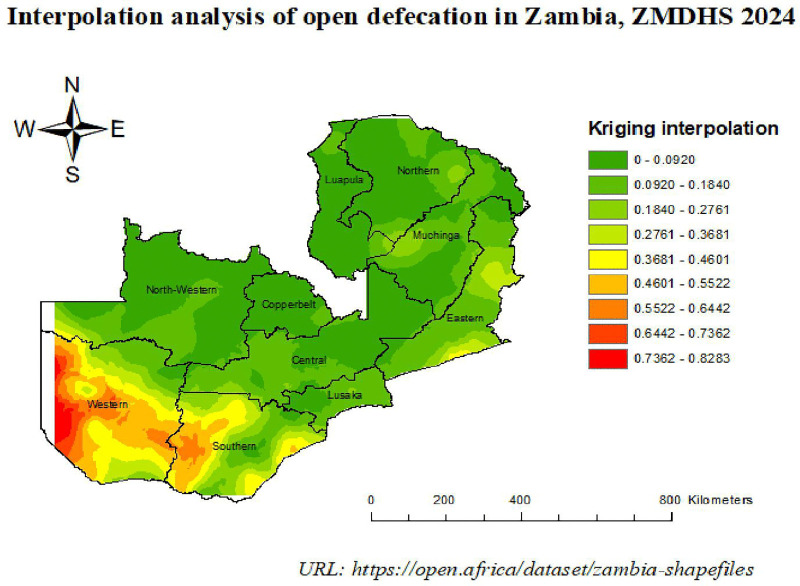
Kriging interpolation of open defecation in Zambia, ZMDHS 2024.

To predict open defecation in an unobserved area, this study used ordinary Kriging geostatistical interpolation methods as illustrated in Table 1. Regular kriging interpolation analysis predicted that open defecation would rise from dark green to red areas in Zambia DHS 2024, as seen in Fig 7 below. In the Western and Southern regions, red color denoted areas with high predictions of open defecation practice. In Muchinga, Copperbelt, Luapula, Northern, Central, Northwestern and certain parts of the Lusaka regions, dark green areas represent areas with low predicted levels of open defecation practice.

#### Spatial SaTScan analysis.

**Fig 8:**
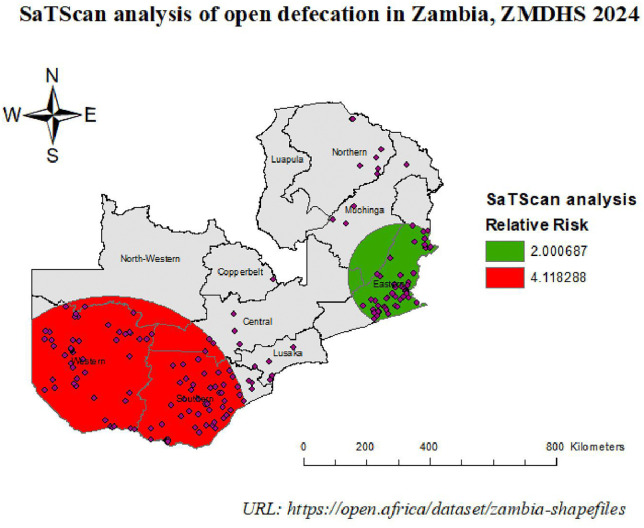
Spatial SaTScan analysis of open defecation practice in Zambia, ZMDHS 2024.

Among 545 clusters, 153 were statistically significant clusters of open defecation practice, which were categorized into primary and secondary clusters. The primary clusters were 104 statistically significant clusters observed in Western, Northwestern, Southern and Central regions of Zambia and centered at −17.4653N, 24.2384E, 412.1528 km with an LLR of 419.10, RR: 4.12, at p-value less than 0.001. It indicated that households inside the spatial window had 4.12 times higher risk of open defecation practice than households outside the window. The secondary clusters were 49 statistically significant clusters and observed in Eastern, Muchinga and some part of Central and centered at −13.1838N, 32.5030E, 163.8676 km with an LLR of 51.85, RR: 1.60, at p-value less than 0.001. It indicated that households inside the spatial window had a 2.0 times higher risk of open defecation practice than households outside the window as shown in Table 3 and Fig 8.

**Table 3:** Significant clusters of open defecation practice in Zambia, ZMDHS 2024.

Cluster	#location	#population	#case	RR	LLR	Coordinates/Radius	P-value
**Primary**	104	2,349	742	4.12	419.10	−17.4653N, 24.2384E, 412.1528 km	<0.001
**Secondary**	49	1,164	258	2.00	51.85	−13.1838N, 32.5030E, 163.8676 km	<0.001

### Machine learning analysis

#### Class distribution.

**Fig 9:**
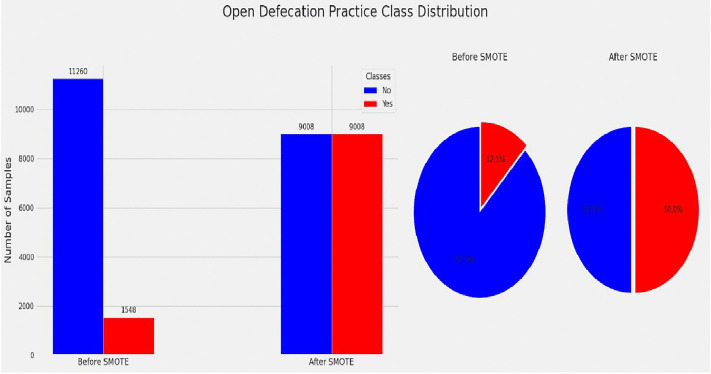
Comparison of open defecation practice before and after SMOTE in Zambia, ZMDHS 2024.

Among all study participants (N = 12808), the distribution of open defecation was evaluated. According to the analysis as shown in Fig 9, the classes were distributed with 1,548 (12.1%) respondents reporting open defecation practices (Yes) and 11,260 (87.9%) respondents reporting no open defecation practices (No). The dataset showed a significant class imbalance because the distribution of the two categories was not proportional. From the balanced training dataset, the SMOTE generated 7,460 additional synthetic observations on the minority group to balance the unbalanced class distribution of open defecation practice.

#### Model performance.

Extreme gradient boosting, adaptive boosting, cat boosting, light gradient boosting, random forest, decision tree and logistic regression were the seven machine learning algorithms that were used to predict the practice of open defecation. Model performance was assessed using the AUC, accuracy, precision, recall, and F1 score. Logistic regression algorithm was the best model on unbalanced training dataset, which was 85.92% in AUC and 89.27% in accuracy. Whereas, light gradient boosting algorithm was the best model on the balanced training dataset, which was 83.83% in AUC and 82.05% in accuracy as shown in Table 4. This suggests that light gradient boost delivered the dataset’s most consistent and balanced classification performance, better capturing both positive and negative instances of open defecation than the other methods.

**Table 4:** Comparison of algorithms with different performance matrix of open defecation practice in Zambia, ZMDHS 2024.

Machine learning model	Weight	Performance (%)				
		AUC	Accuracy	Precision	Recall	F1-Score
**Decision Tree**	Unbalanced	66.88	84.19	33.22	30.32	31.70
	Balanced	69.11	82.08	33.48	48.71	39.68
**Random Forest**	Unbalanced	80.46	87.16	45.32	29.68	35.87
	Balanced	80.56	84.23	38.70	51.93	44.35
**Logistic Regression**	Unbalanced	85.92*	89.27*	62.77*	27.74*	38.48*
	Balanced	83.75	80.68	34.76	68.06	46.02
**Light Gradient Boosting**	Unbalanced	84.97	89.11	60.40	29.03	39.22
	Balanced	83.83*	82.05*	36.26*	63.87*	46.26*
**Cat Boosting**	Unbalanced	83.35	88.41	54.55	25.16	34.44
	Balanced	82.50	82.00	34.87	56.13	43.01
**Adaptive Boosting**	Unbalanced	85.55	89.58	70.48	23.87	35.66
	Balanced	83.47	71.94	26.68	75.48	39.42
**Extreme Gradient Boosting**	Unbalanced	83.65	88.64	56.21	27.74	37.15
	Balanced	82.79	82.05	35.69	60.32	44.84

Note: * = best performance of machine learning algorithm.

#### ROC curves for the tested models.

**Fig 10:**
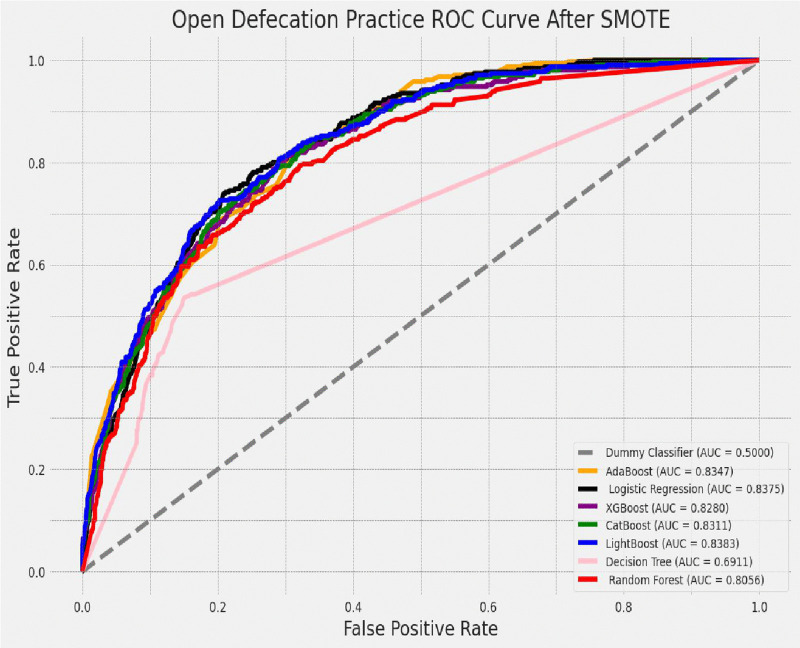
ROC curve of the tested machine learning algorithms of open defecation practice in Zambia, ZMDHS 2024.

The light gradient boosting algorithm was the best model among the seven machine-learning algorithm classifiers used on the balanced training dataset, with an AUC value of 0.8383 for the ROC curve, as shown in Fig 10.

#### Hyperparameter tuning.

**Fig 11:**
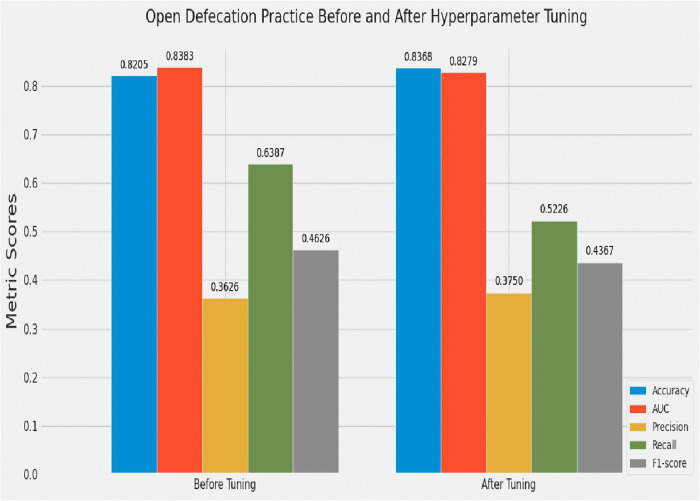
Comparison of before and after tuning with optuna framework of open defecation practice in Zambia, ZMDHS 2024.

The optuna framework hyperparameter tuning did not increase the performance of the balanced training dataset except accuracy and precision. Tuning often restricts depth or splits to avoid overfitting, but in ensemble models, this can reduce diversity and weaken predictive [27]. The optuna hyperparameter search spaces that we used for light gradient boosting were presented in Table 5. The AUC was decreased from 83.83% to 82.79%, as shown in Fig 11. Therefore, the default (before tuning) balanced training dataset was used to test and predict the final model of open defecation practice in Zambia.

**Table 5:** Bayesian optimization optuna framework hyperparameter tuning search space and actual value for light gradient boosting.

Parameters	Default search space	Actual search value
Number of leaves (num_leaves)	20 - 300	127
Maximum depth (max_depth)	3 - 15	14
Learning rate (learning_rate)	1x10^-3^^-^0.3	0.038
Number of trees (n_estimators)	100 - 2000	939
Minimum child samples (min_child_samples)	5 - 100	11
subsample	0.5 - 1	0.716
Column sample before building tree (colsample_bytree)	0.5 - 1	0.523
Range of alpha (reg_alpha)	1x10^-8^ –10	9.3 x10^-5^
Range of lambda (reg_lambda)	1x10^-8^ –10	4.9x10^-8^

#### Prediction of open defecation practice.

**Fig 12:**
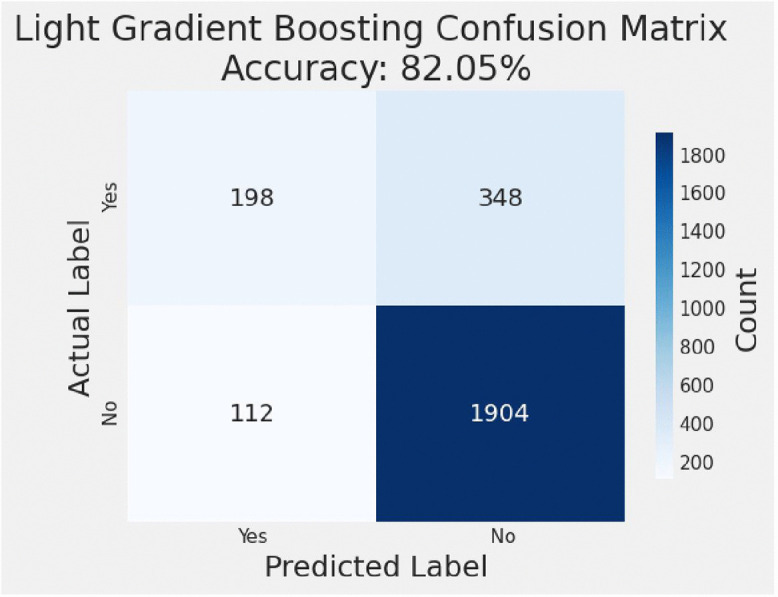
Prediction of open defecation practice using confusion matrix in Zambia, ZMDHS, 2024.

Using a confusion matrix, the predictive performance of the chosen model was assessed. 198 true positives were generated by the model for the classification of open defecation practice, accurately identifying those who reported engaging in this behavior. On the other hand, 348 false negatives were noted, which are instances in which there was open defecation practice, but the model failed to identify it. Regarding the non-open defecation practice side, the model produced 1,904 true negatives, which accurately identified people who did not engage in open defecation practice, and 112 false positives, which showed people who were mistakenly identified as engaging in open defecation practice as illustrated in Fig 12.

#### Global feature importance.

**Fig 13:**
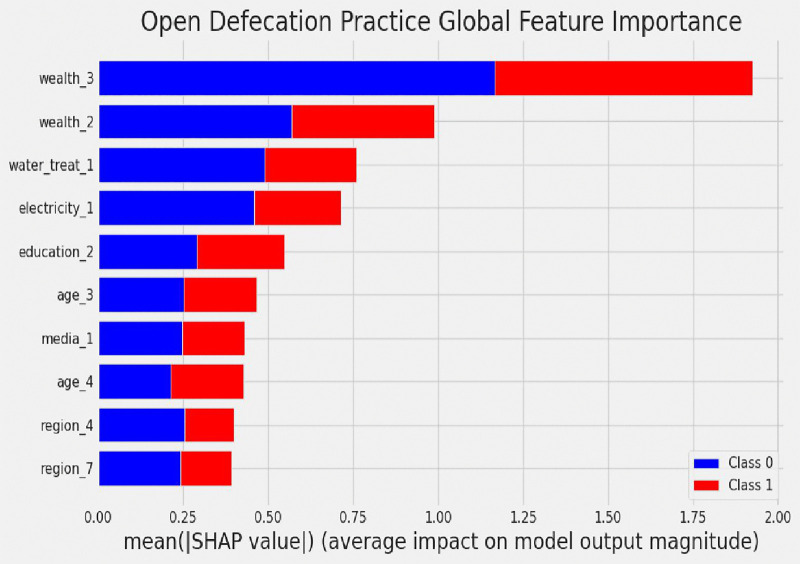
SHAP global feature importance plot of open defecation practice in Zambia, ZMDHS 2024.

To determine the most reliable features associated with classification of open defecation, global feature importance was evaluated using mean absolute SHAP values. Wealth index, access to treated water, access to electricity, educational level, and age of household head, access to media, and region were the most significant associated factors that the model kept according to the analysis presented in Fig 13. Out of all of these, rich and middle wealth index were found to be the most significant associated factors, suggesting that socioeconomic status has a significant influence on the practice of open defecation. Access to treated water was the other significant associated factor linked to the practice of open defecation. Regional-level effects, particularly for respondents from Luapula, and Northern provinces were additional significant associated factor that suggested contextual variations in open defecation practices across regions. A significant contribution was made by educational attainment; secondary education was linked to a decrease in the practice of open defecation, indicating the importance of literacy and awareness in healthcare utilization. Household head aged 46–60 and >60 years old associated factors had significant impact on open defecation practice. Additionally, access to media and electricity were maintained as significant associated factors, highlighting the part that information exposure plays in facilitating the practice of open defecation.

wealth_3 = rich wealth index, wealth_2 = middle wealth index, water_treat_1 = access to treated water, electricity_1 = access to electricity, education_2 = secondary education level, age_3 = age of household head from 46–60, years, age_4 = age of household head >60 years, media_1 = access to media, region_7 = Northern, and region_4 = Luapula,

#### Model interpretation/explanation.

**Fig 14:**
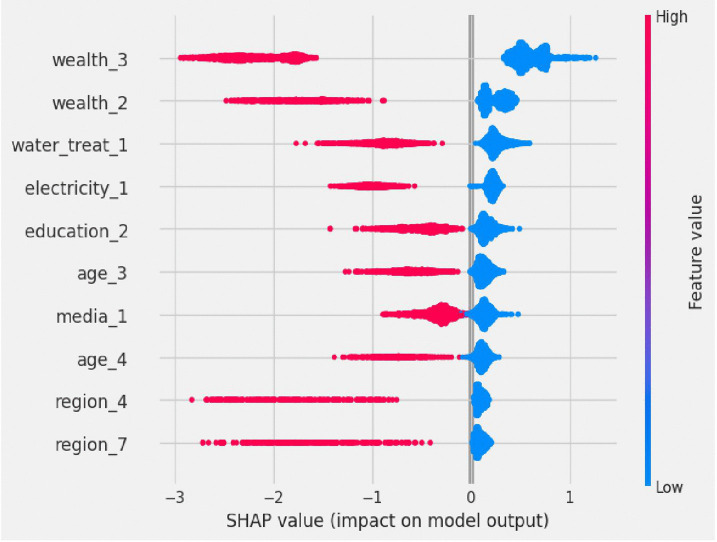
Beeswarm plot ranked by mean absolute SHAP value of open defecation practice in Zambia, ZMDHS 2024.

Differential impacts of important associated factors on the practice of open defecation were brought to light by the SHAP beeswarm plot analysis. SHAP impact (on model output) was used to determine each feature’s contribution to the classification for open defecation practices and model accuracy. Red-dot associated factors have a high predictive probability or push the prediction higher; blue-dot associated factors at the bottom of the tree explainer have a low predictive probability or push the prediction lower. The likelihood of engaging in open defecation was consistently found to be significantly lower among household who were part of the rich and middle wealth index groups; lived in Northern, and Luapula; had secondary levels of education; household head aged 46–60 and >60 years old; and had access to treated water, media, and electricity as shown in Fig 14. These characteristics served as protective associated factors, highlighting the vital roles that financial coverage, education, socioeconomic advantage, and information exposure play in promoting healthcare and environmental sanitation and decrease the practice of open defecation in Zambia.

wealth_3 = rich wealth index, wealth_2 = middle wealth index, water_treat_1 = access to treated water, electricity_1 = access to electricity, education_2 = secondary education level, age_3 = age of household head from 46–60, years, age_4 = age of household head >60 years, media_1 = access to media, region_7 = Northern, and region_4 = Luapula,

## Discussion

The practice of open defecation raises serious public health issues because it contributes to the spread of disease and affects social and environmental aspects [3]. This study aimed to geospatial variation and machine learning approaches to classify open defecation in Zambia. The magnitude of open defecation practice in Zambia was 12.1%. It was less than studies conducted in Ghana 51% [15] and Benin 61% [28], Nigeria 72.7% [29], Chad 69% [30], Ethiopia 32.23% [12], Kenya 44% [31], and Senegal 12.4% [32]. However, it was less than studies conducted in India 5.7% [33] and Nigeria 12% [34]. The variation in open defecation rates could be associated with differences in implementation frameworks, socio-economic statuses, and study design approaches [21]. The reason for this could also be the different government commitments and involvement in community-based initiatives that employ more effective strategies to curb open defecation and accomplish the desired sanitation program [15].

This study’s spatial autocorrelation revealed a high and statistically significant clustering pattern of open defecation practices in Zambia with a Z-score of 5.042 and a Moran’s Index of 0.305 (p-value less than 0.001). Southern, Western and Eastern regions of Zambia were found to have statistically significant hot spot areas of open defecation practice according to hot spot analysis. In order to reduce disparities through evidence-based targeted interventions, hot spot detection facilitates community-based initiatives to raise awareness of open defecation practices. This approach also directs healthcare infrastructure and strategic policymaking [35]. The highly classified likelihood of open defecation in Zambia was observed in Western and Southern regions. Health authorities can allocate resources, gain a better understanding of spatial disparities and alert policymakers to areas that need intervention by using spatial prediction to identify high open defecation practice areas [20]. On the spatial SaTScan analysis, the primary clusters were 104 statistically significant clusters observed in Western, Northwestern, Southern and Central regions of Zambia and centered at −17.4653N, 24.2384E, 412.1528 km with an LLR of 419.10, RR: 4.12, at p-value less than 0.001. It indicated that households inside the spatial window had 4.12 times higher risk of open defecation practice than households outside the window.

For classification purposes, a balanced training dataset was used to train seven supervised machine-learning algorithms. These algorithms included extreme gradient boost, adaptive boost, cat boost, light gradient boost, random forest, decision tree and logistic regression. These seven machine-learning models: AUC, accuracy, precision recall and f1-score were used to compare their performance. Among the algorithms tested on balanced training dataset, light gradient boost achieved the highest AUC score with 83.83% outperforming all other models on the test dataset. However random forest algorithm was the best model for studies conducted in SSA [4]. The difference might be due to the amount of dataset used, study period and the previous study did not include light gradient boosting. For the classification of open defecation practice, the model produced 198 true positives were generated by the model for the classification of open defecation practice, accurately identifying those who reported engaging in this behavior. On the other hand, 348 false negatives were noted, which are instances in which there was open defecation practice, but the model failed to identify it. Regarding the non-open defecation practice side, the model produced 1,904 true negatives, which accurately identified people who did not engage in open defecation practice, and 112 false positives, which showed people who were mistakenly identified as engaging in open defecation practice. According to SHAP value feature importance, wealth index, access to treated water, access to electricity, educational level, and age of household head, access to media, and region were the most important features associated with classification of open defecation practice. Thus, this study can help Zambia achieve Sustainable Development Goal 6 Target 2, which is to prevent human interactions with fecal pathogens in order to lower disease incidences by 2030 [4, 36]. It can also improve policy formulation and optimize resource allocation.

This study showed that Zambia’s regions had varying degrees of association with open defecation practice. Household heads from Northern and Luapula regions of Zambia were observed to be less likely to practice open defecation compared to those from Central region. The geographic variation may be caused by the fact that open defecation rates are higher in areas with poorer infrastructure, less access to clean water, and lower household incomes, while they are significantly lower in areas with greater government funding for sanitation initiatives [37]. Because they influence the strength of public health campaigns, the availability of sanitation facilities, and cultural attitudes regarding toilet use, regional variations are significant in determining whether open defecation continues or decreases.

According to this study, the wealth index was an associated factor in the classification of open defecation practice in Zambia. Households in the middle and rich classes were observed to be less likely to report open defecation practice compared to households in poverty. This finding is in line with studies conducted in Mozambique [38], Nigeria [39], India [40], Ghana [15], Ethiopia [41] and SSA [12]. Countries with low incomes have been the sites of the majority of open defecation practices [41]. Given the cost of materials, labor, and even land space for toilets, middle-class and wealthy households are more able to afford building or maintaining sanitation facilities such as pit latrines or flush toilets [4]. Wealthy households can afford to provide better restroom facilities for their families, but poor households that are fortunate enough to have toilets typically share them with other households [15]. However, the prevalence of open defecation has been reported to be higher in upper middle‑income countries than in lower middle‑income countries, which contrasts with this research. This discrepancy may be due to smaller sample sizes in upper middle‑income countries compared to lower middle‑income countries [12].

This study found that the age of household head was an associated factor in open defecation practice. Household heads aged 46–60 and above 60 years were observed to be less likely to report open defecation compared to household heads aged 15–30 years. This result is supported by studies conducted in SSA [14], Ethiopia [42], and Ghana [43]. The possible reason might be due to elder household heads may have greater experience and financial resources for managing household infrastructure, including sanitation facilities [43]. In addition, elder individuals may have greater exposure to sanitation education and community health programs, which is associated with better sanitation practices [42]. As people age, they may prefer or adopt behaviors that improve their quality of life [4].

According to this study, educational level was an associated factor in the practice of open defecation. Households with secondary education were observed to be less likely to report open defecation compared to uneducated households. This finding is supported by a studies conducted in Indonesia [44], Ghana [15], Nigeria [39], Ethiopia [41], and SSA [4, 12]. This could be due to the fact that household heads with secondary levels of education are generally more aware of the importance of having sanitary facilities and the consequences of open defecation [12]. Additionally, a secondary level of education increases the likelihood that households will be able to earn income, which helps overcome the primary barrier to building a restroom [45]. Educated households may also be more aware of the dangers of open defecation, which is associated with greater use of sanitation facilities and lower risk of fecal-oral disease transmission [4].

This study found that access to media was an associated factor with classification in open defecation practice. Households with media access were observed to have a lower likelihood of reporting open defecation practice compared to those without media access. This finding is supported by studies conducted in Ghana [15], India [46], Haiti [47], and SSA [4]. A possible explanation is that media exposure helps people internalize the advantages of using sanitation facilities, influences attitudes and behaviors in the home, and increases awareness of the harmful effects of open defecation [47]. When media exposure is limited, there is less information available that could influence sanitation practices and community attitudes [48]. Coordinated national awareness campaigns can be effectively carried out via radio, television, and newspapers. Many people who live in different places can receive messages from these devices at the same time. For spreading information about health hazards and access to sanitary facilities, radio is extremely important, particularly in isolated rural areas with little electrical power [45]. In addition to the messages, television is crucial for demonstration or visualization techniques [15].

This study found that access to electricity was an associated factor in open defecation practice. Households with access to electricity were observed to be less likely to report open defecation practice compared to those without electricity. This finding is supported by studies conducted in Yemen [49] and Nigeria [50], and SSA [4]. A possible explanation is that electricity is closely related to infrastructure information access and socioeconomic development, which may be associated with sanitation practices. Households with electricity are more likely to have higher incomes, better living standards, and higher levels of education, which can facilitate investment in sanitation facilities [4]. Access to the media and the internet, which provide health education and awareness campaigns that deter open defecation, is another benefit of electricity.

According to this study, access to treated water was an associated factor in open defecation practice. Households that had access to treated water were observed to be less likely to practice open defecation compared to households without access to treated water. Access to treated, piped, or nearby safe water may reduce open defecation practice by enabling the construction and consistent use of pour-flush latrines, which are otherwise unfeasible without water [51]. Evidence indicates that improved water access significantly lowers open defecation practice rates, improves hygiene, and is a key driver for upgrading from open defecation /unimproved sanitation to toilets [52]. Access to reliable, safe water also increases handwashing with soap, which is often promoted alongside sanitation upgrades, reducing the tendency to defecate in the open.

### Strength and limitation of the study

The strengths of this study were found in its creative integration of machine learning and geospatial analysis, which produced predictive modeling of open defecation practices in Zambia as well as insights into spatial clustering. While hot spot detection showed notable regional differences, providing compelling evidence for focused interventions. However, the cross-sectional design, which limits the ability to draw conclusions about causality; the use of survey data, which may introduce bias; and regional sample differences, which may affect prediction accuracy, are some of the study’s limitations. Furthermore, cultural norms and the quality of sanitation infrastructure were not adequately captured. All of these advantages and disadvantages point to the study’s usefulness in directing evidence-based sanitation policies while also highlighting areas that require more thorough research in the future.

## Conclusion

With a prevalence of 12.1%, this study shows that open defecation is still a major public health concern in Zambia. It varies by region due to socioeconomic disparities, infrastructure constraints, and variations in government-led sanitation initiatives. Strong clustering patterns were found by spatial analysis, highlighting the need for interventions that are geographically targeted. Hot spots were concentrated in Southern, Western and Eastern regions of Zambia. Open defecation practice was successfully classified by machine learning techniques, especially the light gradient boosting model, which achieved best model and identified important associated factors like wealth index, access to treated water, access to electricity, educational level, and age of household head, access to media, and region. These results emphasize how crucial it is to incorporate geospatial and machine learning techniques into the planning of sanitation policies in order to maximize resource allocation and minimize disparities. Governments and policymakers can utilize these findings to design targeted interventions for reducing open defecation based on the identified gaps and disparities. While the results provide actionable evidence for future research should adopt mixed-methods approaches to incorporate cultural and qualitative dimensions, ensuring that social norms and traditional beliefs are adequately represented. Additionally, longitudinal studies are needed to explore how climate change may exacerbate sanitation infrastructure vulnerabilities, thereby linking behavioral insights with environmental resilience. In order to reduce exposure to fecal pathogens and improve public health outcomes by 2030, Zambia can accelerate progress toward SDG 6.2 by addressing socioeconomic and infrastructural barriers and utilizing digital awareness campaigns.

## Abbreviations

AUC: Area under the Curve
DHS: Demographic Health Survey
SDG: Sustainable Development Goal
SHAP: Shapely Additive Explanation
SMOTE: Synthetic Minority Oversampling Technique
SSA: Sub-Saharan Africa
ZMDHS: Zambia Demographic Health Survey.
