# Supplementary material for: Geospatial variation and machine learning approaches to predict open defecation practice in Zambia
Source: PLoS One. 2026 Jun 10;21(6):e0350923. doi: 10.1371/journal.pone.0350923 (PMC13252813; doi:10.1371/journal.pone.0350923)
Supplement: Attachment — Submitted filename: Response to Reviewers.docx [file pone.0350923.s001.docx]

**Date: April** 28, 2025

**Dear editorial board member (s) of** **PLOS One** **journal**

We the authors have been requested to revise the manuscript entitled ***“*Geospatial variation and machine learning approaches to predict open defecation practice in Zambia*”,*** and identified by Manuscript ID PONE-D-26-11222 under **PLOS One** journal, which was submitted for publication. Therefore, we received the editor and reviewer’s revision comments for the betterment of the manuscript before its publication.

We are very thankful for receiving comments, suggestions, directions, questions, and deep insights from the editor and reviewers. We are very happy in receiving constructive and valuable comments for the betterment of the manuscript. Accordingly, we have considered all the comments, questions, directions, and suggestions and provided a point-by-point response letter.

Finally, we have submitted all the required documents in their revised form. We hope that we have addressed all the suggestions, directions, and raised questions and if you believe that point(s) is not addressed, please let us know.

**Thank you very much all editor (s), and reviewers**

**On behalf of the authors**

**Yours sincerely,**

**Andualem Addisu Birlie (Correspondence author)**

**Point-by-point response letter**

**Editor**

**Authors Response:** Muammar Qadafi (Academic Editor), we thank you for recommending us read and respond to all the peer reviewer comments and requesting us to revise our work for its betterment. We hope that we have addressed all the mentioned comments, questions, and suggestions that were raised by the reviewers. All the required documents (revised manuscript, tracked manuscript, and point-by-point response) also have been submitted. If there is any issue that is not addressed, please let us know!!

Thank you so much!!

**Editorial Comments to Author**

**Editor Comment 1 (Style requirements)**

**Authors’ response:** We acknowledge PLOS One’s style requirements, including those for file naming and formatting. The manuscript has been carefully revised to conform to the journal’s style templates. All files have been renamed and structured according to PLOS One’s guidelines, ensuring consistency, clarity, and compliance with submission standards.

**Editor Comment 2 (Code sharing)**

**Authors’ response:** We acknowledge PLOS One’s guidelines on code sharing. All author‑generated code underpinning the findings has been prepared for release and made available in a stable public repository without restrictions, following best practices to ensure reproducibility and reuse on GitHub (<https://github.com/ayabzmam/Open-Defecation/tree/main> )

**Editor Comment 3 (Data repository)**

**Authors’ response:** Thank you for noting this. We have uploaded the minimal dataset necessary to replicate our findings to an acceptable public repository and updated the Data Availability statement with the relevant URL on <https://dhsprogram.com/data/dataset_admin/index.cfm>.

**Editor Comment 4 (Figures and copyright)**

**Authors’ response:** We confirm that all figures, including maps and workflow diagrams, are original and owned by the authors. No copyrighted or proprietary sources (e.g., Google Maps, Earth, or Street View) were used. The initial blurriness was due to TIF format conversion; we have replaced all figures with high‑resolution versions and improved legends, labels, and cartographic design to meet publication standards under CC BY 4.0.

**Editor Comment 5 (Table reference)**

**Authors’ response:** Thank you for pointing this out. We have revised the manuscript to explicitly reference Table 2 in the Results section, ensuring that readers are directed to the table and that production can properly link it.

**Reviewer comments to Author**

**Reviewer #1:**

**Concern#1**: The outcome variable was incorrectly defined, conflating open defecation with unimproved sanitation. This likely inflated prevalence and affected downstream results. The variable must be redefined according to DHS/JMP standards and the analysis rerun.

**Authors’ response:** Thank you for this important observation. We have redefined the outcome variable strictly according to DHS/JMP sanitation categories, ensuring that only households reporting “no facility or defecating in bush or field” are classified as practicing open defecation. Following this correction, we reran the entire analysis, and the updated prevalence is 12.1%. All spatial and machine‑learning results have been revised accordingly. This major revision improves the methodological rigor and accuracy of the study.

**Concern#2**: The manuscript does not clearly state whether the spatial analyses use individual households, DHS clusters, or aggregated regional values. Specifically, DHS GPS data are provided at the cluster level and are spatially displaced for confidentiality. If household records were assigned repeated cluster coordinates and then analyzed as point data, Moran’s I, hotspot analysis, and SaTScan results may be artificially inflated. Therefore, the authors must clearly state: (1) the unit of spatial analysis; (2) the number of spatial points/clusters used; (3) whether outcome prevalence was aggregated to the cluster level; (4) and what spatial weights or distance band specification was used.

**Authors’ response:** Thank you for this important clarification. We have revised the Methods section to clearly state that: The unit of spatial analysis was the DHS cluster level; A total of 545 clusters were included; Outcome prevalence was aggregated to the cluster level and An adaptive bandwidth specification was used for spatial weights.

**Concern#3**: The use of ordinary kriging to interpolate open defecation practice needs more methodological support. Kriging assumes particular spatial properties that are not demonstrated here, and the manuscript does not report the semivariogram model, cross‑validation performance, or prediction uncertainty. If the input is a binary household outcome or cluster‑level proportion derived from displaced DHS points, the assumptions need to be clearly justified.

**Authors’ response:** Thank you for highlighting this methodological issue. We have revised the Methods section to clarify that ordinary kriging was applied to cluster‑level proportions of open defecation. We now report the semivariogram model used, cross‑validation performance, and prediction uncertainty. The assumptions underlying kriging are explicitly justified in relation to the displaced DHS cluster data. These additions strengthen the transparency and validity of the interpolation approach.

**Concern#4**: The machine‑learning section needs clearer reporting. The manuscript compares several tree‑based methods but does not include a simple interpretable baseline such as logistic regression. Without that baseline, it is hard to judge whether using more complex models adds meaningful value. The paper should also report: (1) the full feature set used in each model; (2) the hyperparameter search space; (3) selected tuning parameters; (4) software package versions; (5) decision threshold used for classification; (6) and calibration metrics in addition to discrimination metrics.

**Authors’ response:** Thank you for this valuable feedback. We have revised the manuscript to include logistic regression as a baseline model, provided the complete feature set, reported the hyperparameter search space and selected tuning parameters, specified the software package versions, and stated the decision threshold used for classification. We did not include calibration metrics because the primary aim of this research was classification rather than probability estimation. As you now that calibration metrics is used for measuring probability. These revisions improve transparency and allow clearer evaluation of the benefit of complex models.

**Concern#5**: The paper frequently refers to “determinants” and “predictors” as though causal relationships have been established, but the design is cross‑sectional. The authors should use more cautious language such as “associated factors” or “features associated with classification.” This matters especially in the discussion and conclusion.

**Authors’ response:** Thank you for this important observation. We have carefully revised the manuscript to replace causal terms such as “determinants” and “predictors” with more cautious phrasing, including “associated factors” and “features associated with classification.” These changes have been applied consistently throughout the text, particularly in the discussion and conclusion, to ensure that the language accurately reflects the cross‑sectional design and avoids implying causality.

**Concern#6**: The figures are low resolution and not publication‑ready. This is especially noticeable for the maps and the machine‑learning workflow figure on the later pages. Legends, labels, and cartographic design need improvement.

**Authors’ response:** Thank you for pointing this out. The low resolution was due to the use of TIF format in the initial submission. We have replaced all figures with high‑resolution versions, improved legends and labels, and refined the cartographic design to ensure that the maps and workflow diagrams are publication‑ready.

**Reviewer #2:**

**Concern#1**: This study represents a significant advancement in addressing Zambia's sanitation crisis. Its primary strength lies in the integration of geospatial analysis with artificial intelligence to analyze highly recent (2024) data from over 12,000 households. The paper successfully bridges a critical research gap by moving beyond general national estimates to identify geographically precise "hotspots" and explain complex behavioral drivers using the advanced LightGBM model. Furthermore, the use of SHAP analysis provides transparency by quantifying the influence of key variables such as wealth, education, and digital access—on predicting health behaviors. However, scientific gap remains regarding qualitative and cultural dimensions, as quantitative data alone cannot fully capture the social norms or traditional beliefs that may drive individuals to practice open defecation despite having access to latrines. Additionally, there is a need for future longitudinal research to link these findings with the impact of climate change on regional sanitation infrastructure.

**Authors’ response:** We sincerely thank you for your constructive feedback and recognition of the strengths of our study. In response to your comments, we have revised the **Discussion and Conclusion** sections to explicitly acknowledge the limitations of relying solely on quantitative data and to emphasize the importance of incorporating qualitative and cultural dimensions in future research. We also highlight the need for longitudinal studies to assess how climate change may exacerbate sanitation infrastructure vulnerabilities, thereby linking behavioral insights with environmental resilience. These revisions strengthen the manuscript by addressing the gaps you identified and providing a clearer roadmap for future work.

**With regards!**

**Thank you!**

**Yours sincerely**

**Andualem Addisu Birlie (Correspondence author)**
