# Supplementary material for: Geospatial variation and machine learning approaches to predict open defecation practice in Zambia
Source: PLoS One. 2026 Jun 10;21(6):e0350923. doi: 10.1371/journal.pone.0350923 (PMC13252813; doi:10.1371/journal.pone.0350923)
Supplement: Peer review history

# Peer review: Decision Letter 0

**DOI:** 10.1371/journal.pone.0350923.r001

**Authors:** Muammar Qadafi

19 Apr 2026

PONE-D-26-11222Geospatial Variation and Machine Learning Approaches to Predict Open Defecation Practice in ZambiaPLOS One

Dear Dr. Birlie,

Thank you for submitting your manuscript to PLOS ONE. After careful consideration, we feel that it has merit but does not fully meet PLOS ONE’s publication criteria as it currently stands. Therefore, we invite you to submit a revised version of the manuscript that addresses the points raised during the review process.

Please submit your revised manuscript by Jun 03 2026 11:59PM. If you will need more time than this to complete your revisions, please reply to this message or contact the journal office at plosone@plos.org. When you're ready to submit your revision, log on to https://www.editorialmanager.com/pone/ and select the 'Submissions Needing Revision' folder to locate your manuscript file.

Please include the following items when submitting your revised manuscript:

- A letter that responds to each point raised by the academic editor and reviewer(s). You should upload this letter as a separate file labeled 'Response to Reviewers'.
- A marked-up copy of your manuscript that highlights changes made to the original version. You should upload this as a separate file labeled 'Revised Manuscript with Track Changes'.
- An unmarked version of your revised paper without tracked changes. You should upload this as a separate file labeled 'Manuscript'.

If you would like to make changes to your financial disclosure, please include your updated statement in your cover letter. Guidelines for resubmitting your figure files are available below the reviewer comments at the end of this letter.

If applicable, we recommend that you deposit your laboratory protocols in protocols.io to enhance the reproducibility of your results. Protocols.io assigns your protocol its own identifier (DOI) so that it can be cited independently in the future. For instructions see: https://journals.plos.org/plosone/s/submission-guidelines#loc-laboratory-protocols. Additionally, PLOS ONE offers an option for publishing peer-reviewed Lab Protocol articles, which describe protocols hosted on protocols.io. Read more information on sharing protocols at https://plos.org/protocols?utm_medium=editorial-email&utm_source=authorletters&utm_campaign=protocols.

As the corresponding author, your ORCID iD is verified in the submission system and will appear in the published article. PLOS supports the use of ORCID, and we encourage all coauthors to register for an ORCID iD and use it as well. Please encourage your coauthors to verify their ORCID iD within the submission system before final acceptance, as unverified ORCID iDs will not appear in the published article. *Only* the individual author can complete the verification step; PLOS staff *cannot* verify ORCID iDs on behalf of authors.

We look forward to receiving your revised manuscript.

Kind regards,

Muammar Qadafi

Academic Editor

PLOS One

Journal Requirements:

When submitting your revision, we need you to address these additional requirements.

1. Please ensure that your manuscript meets PLOS ONE's style requirements, including those for file naming. The PLOS ONE style templates can be found at

https://journals.plos.org/plosone/s/file?id=wjVg/PLOSOne_formatting_sample_main_body.pdf and

https://journals.plos.org/plosone/s/file?id=ba62/PLOSOne_formatting_sample_title_authors_affiliations.pdf

2. Please note that PLOS One has specific guidelines on code sharing for submissions in which author-generated code underpins the findings in the manuscript. In these cases, we expect all author-generated code to be made available without restrictions upon publication of the work. Please review our guidelines at https://journals.plos.org/plosone/s/materials-and-software-sharing#loc-sharing-code and ensure that your code is shared in a way that follows best practice and facilitates reproducibility and reuse.

3. Thank you for uploading your study's underlying data set. Unfortunately, the repository you have noted in your Data Availability statement does not qualify as an acceptable data repository according to PLOS's standards.

At this time, please upload the minimal data set necessary to replicate your study's findings to a stable, public repository (such as figshare or Dryad) and provide us with the relevant URLs, DOIs, or accession numbers that may be used to access these data. For a list of recommended repositories and additional information on PLOS standards for data deposition, please see https://journals.plos.org/plosone/s/recommended-repositories.

4. We note that Figures 1, 6, 7 and 8 in your submission contain map images which may be copyrighted. All PLOS content is published under the Creative Commons Attribution License (CC BY 4.0), which means that the manuscript, images, and Supporting Information files will be freely available online, and any third party is permitted to access, download, copy, distribute, and use these materials in any way, even commercially, with proper attribution. For these reasons, we cannot publish previously copyrighted maps or satellite images created using proprietary data, such as Google software (Google Maps, Street View, and Earth). For more information, see our copyright guidelines: http://journals.plos.org/plosone/s/licenses-and-copyright.

We require you to either (1) present written permission from the copyright holder to publish these figures specifically under the CC BY 4.0 license, or (2) remove the figures from your submission:

1. You may seek permission from the original copyright holder of Figure(s) [#] to publish the content specifically under the CC BY 4.0 license.

We recommend that you contact the original copyright holder with the Content Permission Form (http://journals.plos.org/plosone/s/file?id=7c09/content-permission-form.pdf) and the following text:

“I request permission for the open-access journal PLOS ONE to publish XXX under the Creative Commons Attribution License (CCAL) CC BY 4.0 (http://creativecommons.org/licenses/by/4.0/). Please be aware that this license allows unrestricted use and distribution, even commercially, by third parties. Please reply and provide explicit written permission to publish XXX under a CC BY license and complete the attached form.”

Please upload the completed Content Permission Form or other proof of granted permissions as an "Other" file with your submission.

In the figure caption of the copyrighted figure, please include the following text: “Reprinted from [ref] under a CC BY license, with permission from [name of publisher], original copyright [original copyright year].”

2. If you are unable to obtain permission from the original copyright holder to publish these figures under the CC BY 4.0 license or if the copyright holder’s requirements are incompatible with the CC BY 4.0 license, please either i) remove the figure or ii) supply a replacement figure that complies with the CC BY 4.0 license. Please check copyright information on all replacement figures and update the figure caption with source information. If applicable, please specify in the figure caption text when a figure is similar but not identical to the original image and is therefore for illustrative purposes only.

The following resources for replacing copyrighted map figures may be helpful:

USGS National Map Viewer (public domain): http://viewer.nationalmap.gov/viewer/

The Gateway to Astronaut Photography of Earth (public domain): http://eol.jsc.nasa.gov/sseop/clickmap/

Maps at the CIA (public domain): https://www.cia.gov/library/publications/the-world-factbook/index.html and https://www.cia.gov/library/publications/cia-maps-publications/index.html

NASA Earth Observatory (public domain): http://earthobservatory.nasa.gov/

Landsat: http://landsat.visibleearth.nasa.gov/

USGS EROS (Earth Resources Observatory and Science (EROS) Center) (public domain): http://eros.usgs.gov/#

Natural Earth (public domain): http://www.naturalearthdata.com/

5. We note you have included a table to which you do not refer in the text of your manuscript. Please ensure that you refer to Table 2 in your text; if accepted, production will need this reference to link the reader to the Table.

6. If the reviewer comments include a recommendation to cite specific previously published works, please review and evaluate these publications to determine whether they are relevant and should be cited. There is no requirement to cite these works unless the editor has indicated otherwise.

[Note: HTML markup is below. Please do not edit.]

Reviewers' comments:

Reviewer's Responses to Questions

**Comments to the Author**

1. Is the manuscript technically sound, and do the data support the conclusions?

The manuscript must describe a technically sound piece of scientific research with data that supports the conclusions. Experiments must have been conducted rigorously, with appropriate controls, replication, and sample sizes. The conclusions must be drawn appropriately based on the data presented.

Reviewer #1: Partly

Reviewer #2: Yes

**********

2. Has the statistical analysis been performed appropriately and rigorously?

Reviewer #1: N/A

Reviewer #2: Yes

**********

3. Have the authors made all data underlying the findings in their manuscript fully available?

The PLOS Data policy requires authors to make all data underlying the findings described in their manuscript fully available without restriction, with rare exception (please refer to the Data Availability Statement in the manuscript PDF file). The data should be provided as part of the manuscript or its supporting information, or deposited to a public repository. For example, in addition to summary statistics, the data points behind means, medians and variance measures should be available. If there are restrictions on publicly sharing data—e.g. participant privacy or use of data from a third party—those must be specified.

Reviewer #1: Yes

Reviewer #2: Yes

**********

4. Is the manuscript presented in an intelligible fashion and written in standard English?

PLOS ONE does not copyedit accepted manuscripts, so the language in submitted articles must be clear, correct, and unambiguous. Any typographical or grammatical errors should be corrected at revision, so please note any specific errors here.

Reviewer #1: Yes

Reviewer #2: Yes

**********

5. Review Comments to the Author

Please use the space provided to explain your answers to the questions above. You may also include additional comments for the author, including concerns about dual publication, research ethics, or publication ethics. (Please upload your review as an attachment if it exceeds 20,000 characters)

Reviewer #1: Thank you for inviting me to review this study that addresses an important public health and environmental geography question by combining spatial analysis and machine learning to examine open defecation in Zambia using 2024 DHS data. The topic is relevant, the national scope is potentially valuable, and the attempt to combine hotspot mapping with predictive modeling is promising. The manuscript also has policy relevance, especially for geographically targeted WASH interventions. However, the current version has several major methodological and interpretive problems that need to be resolved before it is suitable for publication. Below please see my concerns:

1. First of all, the outcome variable appears to be incorrectly defined. The paper states that “open defecation practice” includes households using pit latrines without slabs/platforms and bucket latrines, in addition to households defecating in fields, bushes, forests, open water, or other open spaces. That is not a standard definition of open defecation. It conflates open defecation with unimproved sanitation. As written, the study is not measuring open defecation alone; it is measuring a broader category of poor sanitation access. This likely inflates the reported prevalence of 46.42% and affects all downstream spatial and machine-learning results. The variable must be redefined according to standard DHS/JMP sanitation categories, and the entire analysis should be rerun.

2. The manuscript does not clearly state whether the spatial analyses use individual households, DHS clusters, or aggregated regional values. Specifically, DHS GPS data are provided at the cluster level and are spatially displaced for confidentiality. If household records were assigned repeated cluster coordinates and then analyzed as point data, Moran’s I, hotspot analysis, and SaTScan results may be artificially inflated. Therefore. The authors must clearly state: (1) the unit of spatial analysis; (2) the number of spatial points/clusters used; (3) whether outcome prevalence was aggregated to the cluster level; (4) and what spatial weights or distance band specification was used.

3. In the methodological section, the use of ordinary kriging to interpolate open defecation practice needs much more methodological support. Kriging assumes particular spatial properties that are not demonstrated here, and the manuscript does not report the semivariogram model, cross-validation performance, or prediction uncertainty. If the input is a binary household outcome or cluster-level proportion derived from displaced DHS points, the assumptions need to be clearly justified.

4. In addition, the machine-learning section also needs clearer reporting. The machine-learning analysis is potentially useful, but it is not yet sufficiently transparent. The manuscript compares several tree-based methods, but does not include a simple interpretable baseline such as logistic regression. Without that baseline, it is hard to judge whether using more complex models adds meaningful value. The paper should also report: (1) the full feature set used in each model; (2) the hyperparameter search space; (3) selected tuning parameters; (4) software package versions; (5) decision threshold used for classification; (6) and calibration metrics in addition to discrimination metrics.

5. The paper frequently refers to “determinants” and “predictors” as though causal relationships have been established, but the design is cross-sectional. The authors should use more cautious language such as “associated factors” or “features associated with classification.” This matters especially in the discussion and conclusion.

Minor comments:

The figures are low resolution and not publication-ready. This is especially noticeable for the maps and the machine-learning workflow figure on the later pages. Legends, labels, and cartographic design need improvement.

Reviewer #2: This study represents a significant advancement in addressing Zambia's sanitation crisis. Its primary strength lies in the integration of geospatial analysis with artificial intelligence to analyze highly recent (2024) data from over 12,000 households. The paper successfully bridges a critical research gap by moving beyond general national estimates to identify geographically precise "hotspots" and explain complex behavioral drivers using the advanced LightGBM model. Furthermore, the use of SHAP analysis provides transparency by quantifying the influence of key variables—such as wealth, education, and digital access—on predicting health behaviors. However, a scientific gap remains regarding qualitative and cultural dimensions, as quantitative data alone cannot fully capture the social norms or traditional beliefs that may drive individuals to practice open defecation despite having access to latrines. Additionally, there is a need for future longitudinal research to link these findings with the impact of climate change on regional sanitation infrastructure.

**********

6. PLOS authors have the option to publish the peer review history of their article (what does this mean?). If published, this will include your full peer review and any attached files.

If you choose “no”, your identity will remain anonymous but your review may still be made public.

**Do you want your identity to be public for this peer review?** For information about this choice, including consent withdrawal, please see our Privacy Policy.

Reviewer #1: No

Reviewer #2: **Yes:** Dr.Raheem Al-Abdan

**********

[NOTE: If reviewer comments were submitted as an attachment file, they will be attached to this email and accessible via the submission site. Please log into your account, locate the manuscript record, and check for the action link "View Attachments". If this link does not appear, there are no attachment files.]

To ensure your figures meet our technical requirements, please review our figure guidelines: https://journals.plos.org/plosone/s/figures

You may also use PLOS’s free figure tool, NAAS, to help you prepare publication quality figures: https://journals.plos.org/plosone/s/figures#loc-tools-for-figure-preparation.

NAAS will assess whether your figures meet our technical requirements by comparing each figure against our figure specifications.

# Author comment: Author response to Decision Letter 1

**DOI:** 10.1371/journal.pone.0350923.r002

28 Apr 2026

Editorial Comments to Author

Editor Comment 1 (Style requirements)

Authors’ response: We acknowledge PLOS One’s style requirements, including those for file naming and formatting. The manuscript has been carefully revised to conform to the journal’s style templates. All files have been renamed and structured according to PLOS One’s guidelines, ensuring consistency, clarity, and compliance with submission standards.

Editor Comment 2 (Code sharing)

Authors’ response: We acknowledge PLOS One’s guidelines on code sharing. All author generated code underpinning the findings has been prepared for release and made available in a stable public repository without restrictions, following best practices to ensure reproducibility and reuse on GitHub (https://github.com/ayabzmam/Open-Defecation/tree/main )

Editor Comment 3 (Data repository)

Authors’ response: Thank you for noting this. We have uploaded the minimal dataset necessary to replicate our findings to an acceptable public repository and updated the Data Availability statement with the relevant URL on https://dhsprogram.com/data/dataset_admin/index.cfm.

Editor Comment 4 (Figures and copyright)

Authors’ response: We confirm that all figures, including maps and workflow diagrams, are original and owned by the authors. No copyrighted or proprietary sources (e.g., Google Maps, Earth, or Street View) were used. The initial blurriness was due to TIF format conversion; we have replaced all figures with high resolution versions and improved legends, labels, and cartographic design to meet publication standards under CC BY 4.0.

Editor Comment 5 (Table reference)

Authors’ response: Thank you for pointing this out. We have revised the manuscript to explicitly reference Table 2 in the Results section, ensuring that readers are directed to the table and that production can properly link it.

Reviewer comments to Author

Reviewer #1:

Concern#1: The outcome variable was incorrectly defined, conflating open defecation with unimproved sanitation. This likely inflated prevalence and affected downstream results. The variable must be redefined according to DHS/JMP standards and the analysis rerun.

Authors’ response: Thank you for this important observation. We have redefined the outcome variable strictly according to DHS/JMP sanitation categories, ensuring that only households reporting “no facility or defecating in bush or field” are classified as practicing open defecation. Following this correction, we reran the entire analysis, and the updated prevalence is 12.1%. All spatial and machine learning results have been revised accordingly. This major revision improves the methodological rigor and accuracy of the study.

Concern#2: The manuscript does not clearly state whether the spatial analyses use individual households, DHS clusters, or aggregated regional values. Specifically, DHS GPS data are provided at the cluster level and are spatially displaced for confidentiality. If household records were assigned repeated cluster coordinates and then analyzed as point data, Moran’s I, hotspot analysis, and SaTScan results may be artificially inflated. Therefore, the authors must clearly state: (1) the unit of spatial analysis; (2) the number of spatial points/clusters used; (3) whether outcome prevalence was aggregated to the cluster level; (4) and what spatial weights or distance band specification was used.

Authors’ response: Thank you for this important clarification. We have revised the Methods section to clearly state that: The unit of spatial analysis was the DHS cluster level; A total of 545 clusters were included; Outcome prevalence was aggregated to the cluster level and An adaptive bandwidth specification was used for spatial weights.

Concern#3: The use of ordinary kriging to interpolate open defecation practice needs more methodological support. Kriging assumes particular spatial properties that are not demonstrated here, and the manuscript does not report the semivariogram model, cross validation performance, or prediction uncertainty. If the input is a binary household outcome or cluster level proportion derived from displaced DHS points, the assumptions need to be clearly justified.

Authors’ response: Thank you for highlighting this methodological issue. We have revised the Methods section to clarify that ordinary kriging was applied to cluster level proportions of open defecation. We now report the semivariogram model used, cross validation performance, and prediction uncertainty. The assumptions underlying kriging are explicitly justified in relation to the displaced DHS cluster data. These additions strengthen the transparency and validity of the interpolation approach.

Concern#4: The machine learning section needs clearer reporting. The manuscript compares several tree based methods but does not include a simple interpretable baseline such as logistic regression. Without that baseline, it is hard to judge whether using more complex models adds meaningful value. The paper should also report: (1) the full feature set used in each model; (2) the hyperparameter search space; (3) selected tuning parameters; (4) software package versions; (5) decision threshold used for classification; (6) and calibration metrics in addition to discrimination metrics.

Authors’ response: Thank you for this valuable feedback. We have revised the manuscript to include logistic regression as a baseline model, provided the complete feature set, reported the hyperparameter search space and selected tuning parameters, specified the software package versions, and stated the decision threshold used for classification. We did not include calibration metrics because the primary aim of this research was classification rather than probability estimation. As you now that calibration metrics is used for measuring probability. These revisions improve transparency and allow clearer evaluation of the benefit of complex models.

Concern#5: The paper frequently refers to “determinants” and “predictors” as though causal relationships have been established, but the design is cross sectional. The authors should use more cautious language such as “associated factors” or “features associated with classification.” This matters especially in the discussion and conclusion.

Authors’ response: Thank you for this important observation. We have carefully revised the manuscript to replace causal terms such as “determinants” and “predictors” with more cautious phrasing, including “associated factors” and “features associated with classification.” These changes have been applied consistently throughout the text, particularly in the discussion and conclusion, to ensure that the language accurately reflects the cross sectional design and avoids implying causality.

Concern#6: The figures are low resolution and not publication ready. This is especially noticeable for the maps and the machine learning workflow figure on the later pages. Legends, labels, and cartographic design need improvement.

Authors’ response: Thank you for pointing this out. The low resolution was due to the use of TIF format in the initial submission. We have replaced all figures with high resolution versions, improved legends and labels, and refined the cartographic design to ensure that the maps and workflow diagrams are publication ready.

Reviewer #2:

Concern#1: This study represents a significant advancement in addressing Zambia's sanitation crisis. Its primary strength lies in the integration of geospatial analysis with artificial intelligence to analyze highly recent (2024) data from over 12,000 households. The paper successfully bridges a critical research gap by moving beyond general national estimates to identify geographically precise "hotspots" and explain complex behavioral drivers using the advanced LightGBM model. Furthermore, the use of SHAP analysis provides transparency by quantifying the influence of key variables such as wealth, education, and digital access—on predicting health behaviors. However, scientific gap remains regarding qualitative and cultural dimensions, as quantitative data alone cannot fully capture the social norms or traditional beliefs that may drive individuals to practice open defecation despite having access to latrines. Additionally, there is a need for future longitudinal research to link these findings with the impact of climate change on regional sanitation infrastructure.

Authors’ response: We sincerely thank you for your constructive feedback and recognition of the strengths of our study. In response to your comments, we have revised the Discussion and Conclusion sections to explicitly acknowledge the limitations of relying solely on quantitative data and to emphasize the importance of incorporating qualitative and cultural dimensions in future research. We also highlight the need for longitudinal studies to assess how climate change may exacerbate sanitation infrastructure vulnerabilities, thereby linking behavioral insights with environmental resilience. These revisions strengthen the manuscript by addressing the gaps you identified and providing a clearer roadmap for future work.

Attachment Submitted filename: Response to Reviewers.docx

# Peer review: Decision Letter 1

**DOI:** 10.1371/journal.pone.0350923.r003

**Authors:** Muammar Qadafi

20 May 2026

Geospatial variation and machine learning approaches to predict open defecation practice in Zambia

PONE-D-26-11222R1

Dear Dr. Birlie,

We’re pleased to inform you that your manuscript has been judged scientifically suitable for publication and will be formally accepted for publication once it meets all outstanding technical requirements.

Within one week, you’ll receive an e-mail detailing the required amendments. When these have been addressed, you’ll receive a formal acceptance letter and your manuscript will be scheduled for publication.

An invoice will be generated when your article is formally accepted. Please note, if your institution has a publishing partnership with PLOS and your article meets the relevant criteria, all or part of your publication costs will be covered. Please make sure your user information is up-to-date by logging into Editorial Manager at Editorial Manager® and clicking the ‘Update My Information' link at the top of the page. For questions related to billing, please contact billing support.

If your institution or institutions have a press office, please notify them about your upcoming paper to help maximize its impact. If they’ll be preparing press materials, please inform our press team as soon as possible -- no later than 48 hours after receiving the formal acceptance. Your manuscript will remain under strict press embargo until 2 pm Eastern Time on the date of publication. For more information, please contact onepress@plos.org.

Kind regards,

Muammar Qadafi

Academic Editor

PLOS One

Additional Editor Comments (optional):

Reviewers' comments:

Reviewer's Responses to Questions

**Comments to the Author**

1. If the authors have adequately addressed your comments raised in a previous round of review and you feel that this manuscript is now acceptable for publication, you may indicate that here to bypass the “Comments to the Author” section, enter your conflict of interest statement in the “Confidential to Editor” section, and submit your "Accept" recommendation.

Reviewer #1: All comments have been addressed

**********

2. Is the manuscript technically sound, and do the data support the conclusions?

The manuscript must describe a technically sound piece of scientific research with data that supports the conclusions. Experiments must have been conducted rigorously, with appropriate controls, replication, and sample sizes. The conclusions must be drawn appropriately based on the data presented.

Reviewer #1: Yes

**********

3. Has the statistical analysis been performed appropriately and rigorously?

Reviewer #1: Yes

**********

4. Have the authors made all data underlying the findings in their manuscript fully available?

The PLOS Data policy requires authors to make all data underlying the findings described in their manuscript fully available without restriction, with rare exception (please refer to the Data Availability Statement in the manuscript PDF file). The data should be provided as part of the manuscript or its supporting information, or deposited to a public repository. For example, in addition to summary statistics, the data points behind means, medians and variance measures should be available. If there are restrictions on publicly sharing data—e.g. participant privacy or use of data from a third party—those must be specified.

Reviewer #1: Yes

**********

5. Is the manuscript presented in an intelligible fashion and written in standard English?

PLOS ONE does not copyedit accepted manuscripts, so the language in submitted articles must be clear, correct, and unambiguous. Any typographical or grammatical errors should be corrected at revision, so please note any specific errors here.

Reviewer #1: Yes

**********

6. Review Comments to the Author

Please use the space provided to explain your answers to the questions above. You may also include additional comments for the author, including concerns about dual publication, research ethics, or publication ethics. (Please upload your review as an attachment if it exceeds 20,000 characters)

Reviewer #1: (No Response)

**********

7. PLOS authors have the option to publish the peer review history of their article (what does this mean?). If published, this will include your full peer review and any attached files.

If you choose “no”, your identity will remain anonymous but your review may still be made public.

**Do you want your identity to be public for this peer review?** For information about this choice, including consent withdrawal, please see our Privacy Policy.

Reviewer #1: No

**********

# Editor report: Acceptance letter

**DOI:** 10.1371/journal.pone.0350923.r004

**Authors:** Muammar Qadafi

PONE-D-26-11222R1

PLOS One

Dear Dr. Birlie,

I'm pleased to inform you that your manuscript has been deemed suitable for publication in PLOS One. Congratulations! Your manuscript is now being handed over to our production team.

At this stage, our production department will prepare your paper for publication. This includes ensuring the following:

* All references, tables, and figures are properly cited

* All relevant supporting information is included in the manuscript submission,

* There are no issues that prevent the paper from being properly typeset

You will receive further instructions from the production team, including instructions on how to review your proof when it is ready. Please keep in mind that we are working through a large volume of accepted articles, so please give us a few days to review your paper and let you know the next and final steps.

Lastly, if your institution or institutions have a press office, please let them know about your upcoming paper now to help maximize its impact. If they'll be preparing press materials, please inform our press team within the next 48 hours. Your manuscript will remain under strict press embargo until 2 pm Eastern Time on the date of publication. For more information, please contact onepress@plos.org.

You will receive an invoice from PLOS for your publication fee after your manuscript has reached the completed accept phase. If you receive an email requesting payment before acceptance or for any other service, this may be a phishing scheme. Learn how to identify phishing emails and protect your accounts at https://explore.plos.org/phishing.

If we can help with anything else, please email us at customercare@plos.org.

Thank you for submitting your work to PLOS ONE and supporting open access.

Kind regards,

PLOS ONE Editorial Office Staff

on behalf of

Dr. Muammar Qadafi

Academic Editor

PLOS One
